# PRIMED: predicting DNA binding residues by leveraging pre-trained protein language models

**DOI:** 10.3389/frai.2026.1763313

**Published:** 2026-03-25

**Authors:** Luoshu Zhang, Xin Li, Ruocen Song, Qianqian Song, Xiao Fan

**Affiliations:** 1 J. Crayton Pruitt Family Department of Biomedical Engineering, University of Florida, Gainesville, FL, United States; 2 Department of Health Outcomes and Biomedical Informatics, University of Florida, Gainesville, FL, United States

**Keywords:** DNA-binding protein, DNA-binding residue, protein language model, supervised machine learning, transfer learning

## Abstract

**Introduction:**

Protein-DNA interactions are central to gene regulation, genome stability, and disease mechanisms. Identifying DNA-binding residues (DBRs) is critical for structural modeling, protein engineering, and therapeutic design. Although experimental approaches provide valuable insights, they remain low-throughput and resource-intensive. Computational methods offer scalable alternatives by leveraging protein sequential and structural information to predict DBRs.

**Methods:**

We present PRIMED (Protein Residue Inference using Multilayer perceptron for Enhanced DNA-binding predictions), a machine learning framework that integrates protein representations of distinct biochemical and structural properties from three protein language models: ESM-2, ESM-3, and ESM-C. These representations are concatenated and processed by a multilayer perceptron to perform DBR predictions.

**Results:**

PRIMED demonstrated strong performance across three benchmark datasets: Test-46 and Test-129 from a previous study, CLAPE-DB, and Test-10 K, which we curated from UniProtKB/Swiss-Prot. The model achieves an area under the Receiver Operating Characteristic curve (AUC) of 0.92 and a Matthews Correlation Coefficient (MCC) of 0.64 on Test-46, as well as an AUC of 0.93 and MCC of 0.45 on Test-129. On Test-10 K, PRIMED demonstrates generalizability across proteins with varying DBR percentages, maintaining competitive performance relative to the runner-up method, CLAPE-DB.

**Discussion:**

These results highlight the effectiveness of integrating diverse protein language model representations for accurate, transferable DBR predictions.

## 1 Introduction

DNA-binding proteins (DBPs) play a central role in numerous cellular processes, including gene regulation, replication, repair, recombination, and chromatin organization (Takeda et al., 1983). These proteins recognize specific DNA sequential (Qu et al., 2019) or structural motifs (Shanahan et al., 2004) through conserved DNA-binding domains (Lee et al., 1993), such as helix-turn-helix (HTH) (Aravind et al., 2005), zinc fingers (Jantz et al., 2004), leucine zippers (Landschulz et al., 1988), and homeodomains (Laughon, 1991). Dysregulation of DBPs is implicated in diverse diseases (Uryu et al., 2008), including cancer (Shiroma et al., 2020), developmental syndromes (Micucci et al., 2015), and neurodegeneration (Chen-Plotkin et al., 2010). For example, DBP p53 activates DNA damage response genes (Li et al., 2012), while NF-κB (Hayden et al., 2006) and c-Myc (Buggins et al., 2001) regulate immune and proliferative pathways.

A more granular understanding of DNA-binding residues (DBRs) (Schleif, 1988), which are the specific amino acids that mediate direct contacts with DNA, is essential for modeling protein-DNA interactions (Si et al., 2015), engineering DNA-binding specificity (Bogdanove et al., 2018), and annotating functional sites in novel proteins (Nagarajan et al., 2013). Mutations in DBRs may disrupt regulatory networks (Lozada-Chávez et al., 2008), potentially leading to diseases (Pandey and Loscalzo, 2023). Residue-level annotations also support applications in synthetic biology (Gong et al., 2024) and drug design (Xiong et al., 2011).

Experimental techniques such as site-directed mutagenesis (Carter, 1986), electrophoretic mobility shift assays (EMSA) (Hellman and Fried, 2007), chromatin immunoprecipitation followed by sequencing (ChIP-seq) (Song et al., 2016), and DNA affinity purification sequencing (DAP-seq) (Kadonaga and Tjian, 1986) have been instrumental in characterizing DBRs. However, these approaches remain low-throughput, resource-intensive, and difficult to scale to proteome-level annotations (Rastogi et al., 2018). As of June 2023, the UniProt database held approximately 246 million protein sequences (UniProt Consortium, 2019), but fewer than 0.1% had experimentally validated annotations for DBRs (Zhu et al., 2024). In contrast, integrative estimates suggest that 18–36% of human proteins may possess DNA-binding functions (Peled et al., 2016), inferred from domain composition and transcriptional regulatory roles. This gap reflects both a limited experimental throughput and incomplete functional annotation for a significant portion of the human proteome. This observation underscores the urgent need for accurate computational approaches that can operate at the proteome scale (Gromiha and Nagarajan, 2013), enabling the high-throughput prediction of DBPs and their corresponding contact residues.

The evolution of protein digital representations in computational methods reflects a shift from handcrafted, simple features to learned, comprehensive ones. Early approaches relied on a small set, typically 20–30, of manually engineered features, such as physicochemical descriptors, amino acid composition, or position-specific scoring matrices (PSSMs) (Jones, 1999). While these features captured certain predictive patterns, they were insufficient to fully capture the high-dimensional, context-dependent complexity of protein sequences (Wang et al., 2019). Such limited representations failed to capture the nuanced interactions between residues, particularly in diverse, structurally complex proteins like DBPs (Ahmad and Sarai, 2005; Patro et al., 2012; Cetin and Sefer, 2025; Sefer, 2025). To address this, multiple sequence alignments (MSAs) were introduced to incorporate evolutionary context (Rao et al., 2021), offering a richer view of conservation and co-evolutionary patterns (Chatzou et al., 2016). For instance, TargetDBP leveraged MSAs to construct PSSMs that capture conserved sequence motifs critical for identifying DBPs (Hu et al., 2019). However, MSA generation is computationally expensive and sensitive to MSA depth (Chowdhury and Garai, 2017). The emergence of large protein language models (pLMs), such as the Evolutionary Scale Modeling (ESM) family (Lin et al., 2023; Hayes et al., 2025; ESM Team, 2024), marked a paradigm shift.

Unlike manually engineered features or MSA-derived profiles, pLMs trained on millions of proteins across diverse species implicitly capture structural, functional, and evolutionary information without relying on curated annotations or alignment heuristics (Lin et al., 2023). By learning the contextual relationships between amino acids at scale, pLMs encode biochemical and biophysical properties in a data-driven manner, enabling downstream models (Qiu and Wei, 2023) to access rich information about the residue environment (Meier et al., 2021), secondary structure (Chowdhury et al., 2022), binding potential (Zhang and Liu, 2024), and other relevant properties. This not only reduces dependence on domain-specific preprocessing but also enables generalization across previously unseen protein families. As a result, pLM representations are particularly suited for tasks such as DBR prediction, where the scarcity of comprehensive DBR features presents significant challenges. Ultimately, pLM-derived features offer a scalable, unbiased, alignment-free, and biologically informed representation of protein sequences, enabling diverse and generalizable residue-level inference despite their limited direct interpretability (Li et al., 2024).

In this study, we introduce PRIMED (Protein Residue Inference using MLP for Enhanced DNA-binding predictions), a high-throughput machine learning framework for predicting DBRs by integrating complementary representations from multiple pre-trained pLMs (Lin et al., 2023; Hayes et al., 2025; ESM Team, 2024). PRIMED captures diverse biological signals relevant to sequence and structural context, enabling accurate and generalizable residue-level DNA-binding annotation across a broad range of proteins.

## 2 Materials and methods

### 2.1 Datasets for training and evaluation

We used the training datasets from CLAPE-DB (Liu and Tian, 2024), which are based on curated DBP datasets originally introduced by DBPred (Patiyal et al., 2022) and GraphBind (Xia et al., 2021). These datasets contain residue-level annotations derived from experimentally resolved protein-DNA complexes. DBRs were defined as those containing any atom located within 0.5 Å plus the sum of van der Waals radii from any DNA atom. For model evaluation, we used three non-redundant test sets: Test-46 (46 DBPs) and Test-129 (129 DBPs) from CLAPE-DB, and Test-10 K (12,067 DBPs) curated from UniProt/Swiss-Prot. CLAPE-DB test datasets were compiled from previous studies, including GraphBind (Xia et al., 2021), DBPred (Patiyal et al., 2022), BindN+ (Wang et al., 2010), ProNA2020 (Qiu et al., 2020), and PDNA-62 (Ahmad et al., 2004). Residue-level annotations in the test sets followed the same distance-based criterion as those in the training set.

Since most proteins in the previous datasets are from the PDB, we curated a third test dataset from UniProtKB/Swiss-Prot to further assess the model’s generalizability at scale. We constructed this dataset by scanning the entire UniProt database for entries containing either feature annotations with ‘DNA-BIND’ or textual descriptions indicating DNA-binding activity. Residues not annotated as DNA-binding were treated as non-binding. The DBR definition is less stringent compared to the distance-based criteria used in the PDB (Huang et al., 2009), resulting in more continuous binding regions. This difference in annotation standards and diverse protein sequences provides an independent dataset for assessing model generalizability.

To reduce sequence redundancy and prevent information leakage, we performed identifier-based filtering using UniProt-PDB ID mappings (UniProt Consortium, 2015) to ensure that no overlapping proteins from UniProt and PDB were included in our datasets, even though their sequences are not identical (Zaru et al., 2023). We then applied a second round of pairwise sequence similarity filtering within all datasets. Proteins were clustered at 80% sequence identity using MMseqs2 (Steinegger and Söding, 2017), and only a single representative from each protein cluster was retained. This multi-stage filtering process ensured a non-redundant and partition-independent protein corpus.

The resulting datasets consisted of a training set with 1,198 proteins, along with three test sets containing 46, 129, and 12,067 proteins, respectively. A summary of the training and testing dataset compositions, including the percentage of annotated DBRs, is provided in Table 1.

**Table 1:** Summary of training and testing dataset composition.

Subset name	Number of proteins	Number of residues	% binding residues
Training	1,198	470,074	6.35%
Test-46	46	10,876	8.87%
Test-129	129	37,515	5.97%
Test-10 K	12,067	4,874,438	12.98%

To further characterize the residue-level properties of the annotated datasets, we summarized the amino acid composition of DNA-binding residues and compared it with the overall amino acid composition across all residues. The composition in the training set is summarized in Table 2, and summaries for the Test-129, Test-46, and Test-10 K are provided in Supplementary Tables S1–S3. Arginine (R) and lysine (K) are consistently the most enriched residues among DBRs across datasets. Leucine (L) and glutamate (E) are consistently depleted among DBRs relative to their background frequencies.

**Table 2:** Amino acid enrichment and depletion at DNA-binding residues in the training dataset.

Residue	DBR (%)	Background (%)	Enrichment (DBR/background)
R	16.41	5.95	2.76
K	13.16	6.65	1.98
Y	5.07	3.09	1.64
W	1.79	1.12	1.59
H	3.78	2.40	1.58
N	5.74	4.16	1.38
T	6.57	5.15	1.28
S	8.19	7.18	1.14
Q	4.72	4.56	1.04
G	6.33	6.43	0.98
F	3.34	3.68	0.91
M	1.80	2.20	0.82
P	3.10	5.42	0.57
D	2.98	5.31	0.56
A	4.05	7.38	0.55
I	2.79	5.22	0.53
V	3.14	6.09	0.52
C	0.65	1.41	0.46
E	3.05	7.12	0.43
L	3.33	9.48	0.35

DBR (%) denotes the percentage of each amino acid among DNA-binding residues, while Background (%) denotes the percentage among all residues in the dataset. The enrichment ratio was calculated as DBR% divided by Background%, and residues are sorted in descending order of this ratio.

### 2.2 Protein representations

Unlike traditional prediction methods that rely on handcrafted protein features derived from prior human knowledge, we leveraged general, comprehensive representations from pre-trained pLMs, which are free of such biases. These representations can capture novel, previously unseen protein features. In this study, we employed three transformer-based pLMs from the ESM family. These models were trained on millions of protein sequences to capture biologically relevant sequence information, without relying on task-specific objectives, making them well-suited for diverse downstream applications. Specifically, we used the ESM-2 (esm2_t36_3B_UR50D) (Lin et al., 2023), the ESM-3 (ESM3_OPEN_SMALL) (Hayes et al., 2025), and the ESM-C (esmc_600m) (ESM Team, 2024).

Sequences were tokenized and passed through the respective pLMs, and we further trained the amino acid representation for DBR predictions. The representation dimensions for ESM-2, ESM-3, and ESM-C are 2,560, 1,536, and 1,152, respectively. We also concatenated the per-residue representations derived from different ESM models, considering that each model captures complementary aspects of protein properties. Although ESM-2 and ESM-C are both sequence-based protein language models, they differ in architecture, training scale, and optimization strategy, leading to distinct residue-level representations. ESM-2 is trained at a larger scale and is effective at capturing long-range evolutionary and contextual dependencies across protein sequences (Lin et al., 2023). In contrast, ESM-C incorporates updated architectural and training refinements that emphasize efficient local contextual encoding (ESM Team, 2024). ESM-3 further complements these sequence-based representations by incorporating structure-aware pretraining objectives (Hayes et al., 2025), enabling it to encode information related to three-dimensional protein organization. Although ESM-2 and ESM-C are both sequence-based models, differences in their architectures and training regimes yield distinct residue-level representations, whereas ESM-3 provides complementary structure-aware features derived from its structure-focused pretraining. Together, these models capture complementary sequence- and structure-level signals relevant to DNA-binding residue prediction.

### 2.3 Model architecture

We implemented an MLP for DBR predictions using per-residue pLM representation as features. The network comprised fully connected layers with ReLU activations, yielding binding probabilities for each residue through a sigmoid function. We used binary cross-entropy as the loss function, and a dropout rate of 0.5 in all hidden layers to reduce overfitting. Early stopping was applied based on validation loss, with a patience of five epochs and a minimum improvement threshold of 10^−4^ in validation loss. All weights were initialized with default settings.

PLM selection and hyperparameter tuning were performed using a five-fold cross-validation approach. Model evaluation reflects differences in protein representation and architecture. The evaluation metric was the mean area under the Receiver Operating Characteristic (ROC) curve (AUC) across folds. The hyperparameters examined were the number of hidden layers and the dimensionality of each hidden layer. After selecting the optimal hyperparameters, the final model was retrained on the entire training set.

The MLP model computed the output probability $\hat{y}$ through a sequence of nonlinear transformations applied to the input vector x. For an MLP with n hidden layers, the feedforward computation was expressed as:


$$h^{\left(0\right)}=x$$

$$h^{\left(i\right)}=\text{Dropout}(\text{ReLU}(W^{\left(i\right)}h^{\left(i-1\right)}+b^{\left(i\right)})),\text{for}\;i=1,\ldots ,n$$

$$\hat{y}=\sigma (W^{\left(n+1\right)}h^{\left(n\right)}+b^{\left(n+1\right)})$$

Here, $W^{\left(i\right)}$and $b^{\left(i\right)}$ denote the weight matrices and bias vectors of the ith layer; $h^{\left(i\right)}$ represents the hidden state; ReLU(·) is the rectified linear unit activation function; Dropout(·) randomly sets elements to zero with probability p; and σ(·) is the sigmoid activation function used to compute the final probability score.

### 2.4 Evaluation

Model performance was evaluated at the residue level using the following metrics: AUC, Matthew’s correlation coefficient (MCC), sensitivity, specificity, and F1 score. AUC evaluates how well the model’s predicted scores distinguish between binding and non-binding residues, while MCC provides a balanced assessment of binary classification performance, particularly under class imbalance. Let TP, TN, FP, and FN denote the numbers of true positives, true negatives, false positives, and false negatives, respectively. Sensitivity is defined as


$$\text{Sensitivity}=\frac{\mathrm{TP}}{\mathrm{TP}+\mathrm{FN}}$$

and specificity is defined as


$$\text{Specificity}=\frac{\mathrm{TN}}{\mathrm{TN}+\mathrm{FP}}$$

The F1 score is the harmonic mean of precision and recall, given by


$$F1=\frac{2\mathrm{TP}}{2\mathrm{TP}+\mathrm{FP}+\mathrm{FN}}$$

The MCC summarizes binary classification performance by jointly considering all four confusion matrix terms:


$$\mathrm{MCC}=\frac{\mathrm{TP}\cdot \mathrm{TN}-\mathrm{FP}\cdot \mathrm{FN}}{\sqrt{\left(\mathrm{TP}+\mathrm{FP})(\mathrm{TP}+\mathrm{FN})(\mathrm{TN}+\mathrm{FP})(\mathrm{TN}+\mathrm{FN}\right)}}$$

At the same time, the optimal classification threshold was determined to maximize MCC during the final training phase. Evaluation was conducted on the test sets (Test-46, Test-129, and Test-10 K) (see Section 2.1) and compared with several existing methods, including CLAPE-DB (Liu and Tian, 2024), DRNAPred (Yan and Kurgan, 2017), DNAPred (Zhu et al., 2019), SVMnuc (Su et al., 2019), NCBRPred (Zhang et al., 2021), and DBPred (Patiyal et al., 2022). Their evaluation metrics on Test-46 and Test-129 were extracted from the CLAPE-DB study. We also collected the predictions of CLAPE-DB on the Test-10 K dataset and compared them with those of our model to assess the generalization performance.

To assess statistical significance, we performed a paired two-sided *t*-test over ten iterations. In each iteration, AUC scores were computed on 50% of randomly selected test proteins. *p*-values < 0.05 were considered statistically significant.

### 2.5 Feature space visualization and correlation with solvent accessibility

Solvent accessibility has long been recognized as a critical structural determinant for identifying DBRs. Previous methods, such as BindN+ (Wang et al., 2010), DISPLAR (Tjong and Zhou, 2007), DNABind (Liu and Hu, 2013), and PDNAsite (Zhou et al., 2016), have all incorporated predicted or experimentally derived solvent accessibility as a key predictor, demonstrating that surface-exposed residues are more likely to interact with DNA. Building on this insight, we examined whether amino acid representations derived from pre-trained pLMs implicitly encode such structural information. To evaluate this, we calculated the Pearson correlation coefficient between the low-dimensional representation of amino acids and solvent accessibility. Per-residue representations were projected into two dimensions using the Uniform Manifold Approximation and Projection (UMAP) (McInnes et al., 2018). Solvent accessibility was estimated using the Dictionary of Secondary Structure of Proteins (DSSP) algorithm (Kabsch and Sander, 1983), which takes the three-dimensional atomic coordinates from PDB structures as input. We then normalized the solvent accessibility values using min-max scaling to obtain relative solvent accessibility (RSA) values for each protein, which range from 0 (completely buried) to 1 (fully exposed). We visualized the first two UMAP dimensions of Test-129, coloring residues by their RSA values. The protein sequences in Test-46 are not always consistent with their corresponding PDB entries; therefore, they were not included in this analysis.

## 3 Results

### 3.1 Model architecture and hyperparameter selection

We first trained MLPs using protein features from three pLMs: ESM-2, ESM-3, and ESM-C. For each model, we performed five-fold cross-validation on the training dataset to evaluate performance and tune the model’s hyperparameters. Detailed hyperparameter tuning results for the three models are provided in Supplementary Tables S4–S6. We then tested whether their combined representations offer complementary information.

As shown in Table 3, the best-performing architecture consisted of two hidden layers with 256 and 64 units, achieving the highest AUC of 0.960. While single-layer networks achieved comparable but slightly lower performance (AUC in between 0.955 and 0.957), adding a third layer did not further improve accuracy and instead reduced generalization. These results indicate that shallow models may underfit complex patterns, whereas overly deep networks tend to overfit the training data. Supplementary Figure S1 shows cross-entropy loss converging across all five folds. Early stopping was triggered consistently between epochs 5 and 10, confirming that the model converged efficiently without performance degradation. The training loss curves for the three individual pLMs are provided in Supplementary Figures S2–S4.

**Table 3:** The hyperparameter selection for the MLP architecture of PRIMED was evaluated using five-fold cross-validation on the training set.

First layer	Second layer	Third layer	Validation AUC
8	-	-	0.957
64	-	-	0.956
128	-	-	0.956
256	-	-	0.957
512	-	-	0.955
1,024	-	-	0.955
128	64	-	0.958
**256**	**64**	**-**	**0.960**
512	64	-	0.952
512	128	-	0.953
1,024	64	-	0.944
1,024	128	-	0.944
1,024	256	-	0.942
1,536	256	-	0.939
1,024	128	8	0.956
1,024	128	64	0.920
1,024	256	8	0.955
1,024	256	64	0.954
1,024	256	128	0.930

Shown are the averaged AUC across folds for each configuration. The best-performing setting is highlighted in bold.

**Figure 1:**
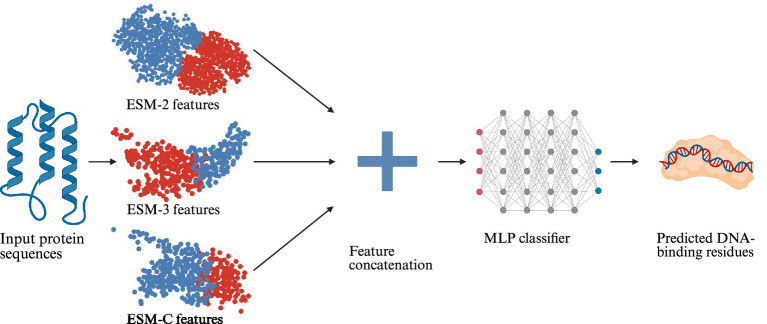
Architecture of PRIMED. Per-residue representations generated by pre-trained ESM language models serve as input to a multilayer perceptron (MLP). The MLP processes each residue’s feature vector through fully connected layers with non-linear activations and outputs a probability score indicating DNA-binding propensity at that position.

As summarized in Table 4, all models demonstrated strong predictive capability, supporting the effectiveness of the representations learned by the pre-trained pLMs. The PRIMED model, which concatenates ESM-2, ESM-3, and ESM-C representations, yielded the most robust predictions on the validation sets (AUC = 0.960, MCC = 0.676). Statistical analyses summarized in Supplementary Table S7 show that PRIMED achieves significantly higher AUC than ESM-2 and ESM-3, while showing no significant difference compared with ESM-C. In terms of MCC, PRIMED is significantly better than ESM-3 and ESM-C but not significantly different from ESM-2. This performance gain suggests that concatenating representations from multiple foundation models captures complementary contextual information, thereby enhancing the model’s discriminative power for classifying DBPs. The overall workflow of the PRIMED model is illustrated in Figure 1.

**Table 4:** DBR prediction performance using different ESM-based model representations.

Model	Validation AUC	Validation MCC
ESM-2	0.954	0.635
ESM-3	0.957	0.522
ESM-C	0.960	0.649
PRIMED	0.960	0.676

Reported values are the mean AUC and MCC across five folds based on the best-performing model parameters.

### 3.2 Prediction performance on benchmark datasets

**Figure 2:**
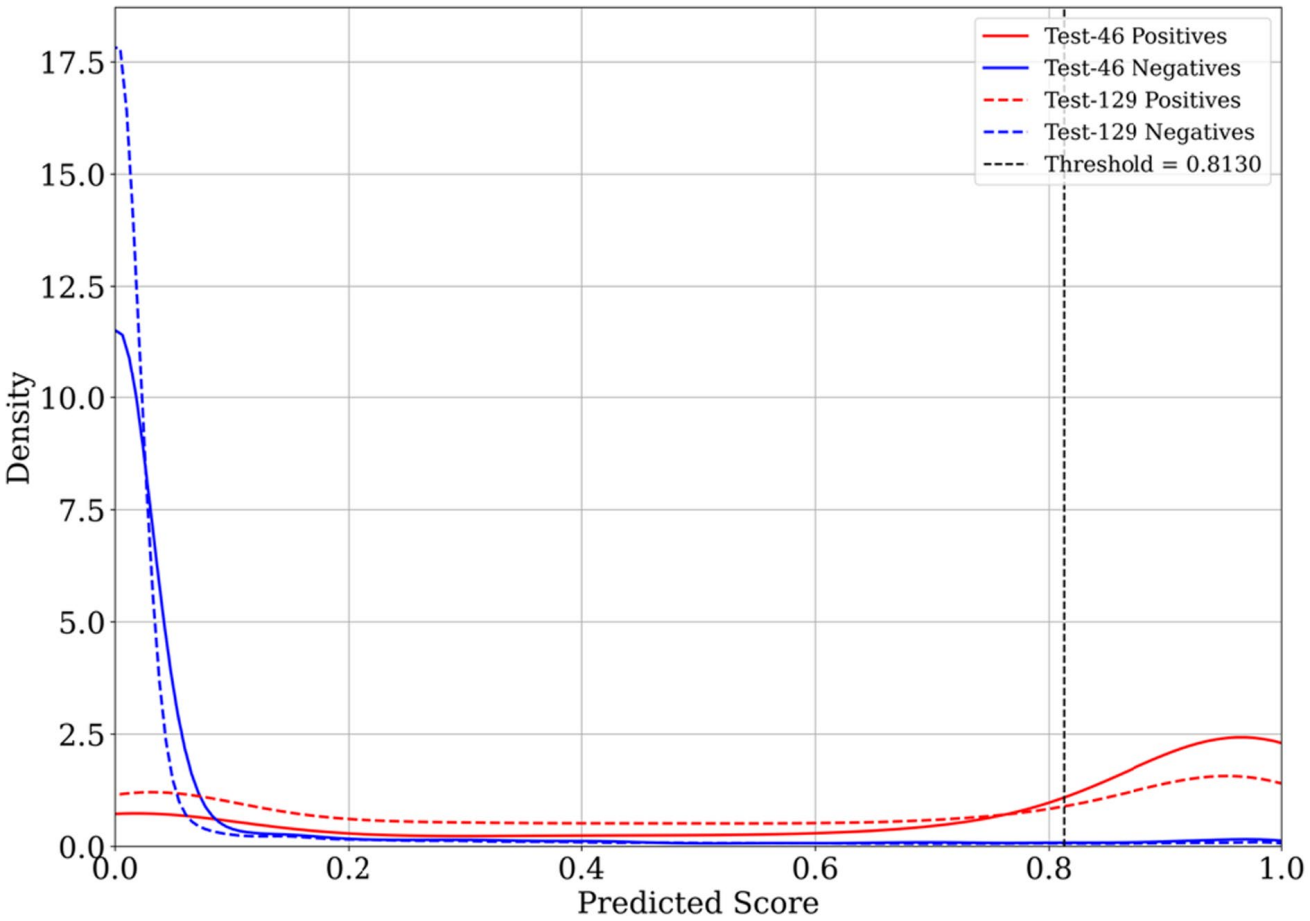
Distribution of predicted residue-level binding scores from PRIMED on Test-46 and Test-129 datasets. Kernel density estimates are shown for binding residues (red lines) and non-binding residues (blue lines), with solid lines representing Test-46 data and dashed lines representing Test-129 data. The vertical dashed line indicates the threshold for binarization as 0.813.

Figure 2 illustrates the distribution of predicted scores, showing clear separation between DNA-binding (positive) and non-binding (negative) residues across Test-46 and Test-129 datasets. The kernel density estimates reveal that negative residues are predominantly concentrated near zero, while positive residues show a broader distribution, with higher scores extending toward and beyond the threshold region. The threshold of 0.813, optimized on the training set, effectively discriminates between the two classes on the test sets, demonstrating the model’s robust classification capability across different test sets.

As shown in Table 5, PRIMED achieves the strongest performance among all sequence-based predictors across both benchmark datasets, consistently attaining the highest AUC and MCC values. On Test-46, PRIMED yields an AUC of 0.919 and an MCC of 0.631, outperforming the second-best model, CLAPE-DB, which scored 0.871 in AUC and 0.401 in MCC. These results correspond to relative improvements of 5.5 and 57%, respectively. Notably, CLAPE-DB achieves higher sensitivity (0.747) than PRIMED (0.631), but this comes at the expense of reduced specificity (0.835 versus 0.975). In contrast, PRIMED maintains a more balanced performance, achieving both high specificity and competitive sensitivity, which translates into more balanced identification of true binding residues while minimizing false positives. The advantages of PRIMED are similarly evident on Test-129. It records an AUC of 0.925 and an MCC of 0.455, compared to 0.881 and 0.389 from CLAPE-DB. This reflects additional gains of 5.0% in AUC and 17% in MCC. Although PRIMED’s sensitivity on Test-129 is modest at 0.375, it achieves the highest specificity among all evaluated methods at 0.985, exceeding that of CLAPE-DB (0.955), SVMnuc (0.966), and NCBRPred (0.969). These findings underscore PRIMED’s strength in precisely distinguishing DBRs from non-binding residues, a property that is especially valuable in large-scale annotation tasks where controlling the false positive rate is critical. These results demonstrate that PRIMED offers a robust and generalizable solution for DBR prediction. Its consistently strong performance across both datasets, especially in terms of specificity and composite metrics such as MCC, highlights its practical advantages over existing methods.

**Table 5:** Residue-level classification performance of PRIMED and other existing methods on two benchmark datasets.

Dataset	Method	Sensitivity	Specificity	F1	AUC	MCC
Test-46	PRIMED	0.631	**0.975**	**0.668**	**0.919**	**0.631**
	CLAPE-DB	**0.747**	0.835	0.434	0.871*	0.401*
	DRNAPred	0.677	0.692	0.291	0.755	0.226
	DNAPred	0.671	0.655	0.254	0.730	0.194
	SVMnuc	0.668	0.666	0.250	0.715	0.192
	NCBRPred	0.677	0.674	0.265	0.713	0.207
	DBPred	0.708	0.784	0.362	0.794	0.320
Test-129	PRIMED	0.375	**0.985**	**0.465**	**0.925**	**0.455**
	CLAPE-DB	**0.464**	0.955	0.427	0.881*	0.389*
	DRNAPred	0.233	0.937	0.210	0.693	0.155
	DNAPred	0.396	0.954	0.373	0.845	0.332
	SVMnuc	0.316	0.966	0.341	0.812	0.304
	NCBRPred	0.312	0.969	0.347	0.823	0.313

Reported are the sensitivity, specificity, F1 score, AUC, and MCC values for each model. * indicate that a statistical test was performed, and PRIMED significantly outperformed the corresponding method. The highest value for each metric within a dataset is shown in bold.

**Figure 3:**
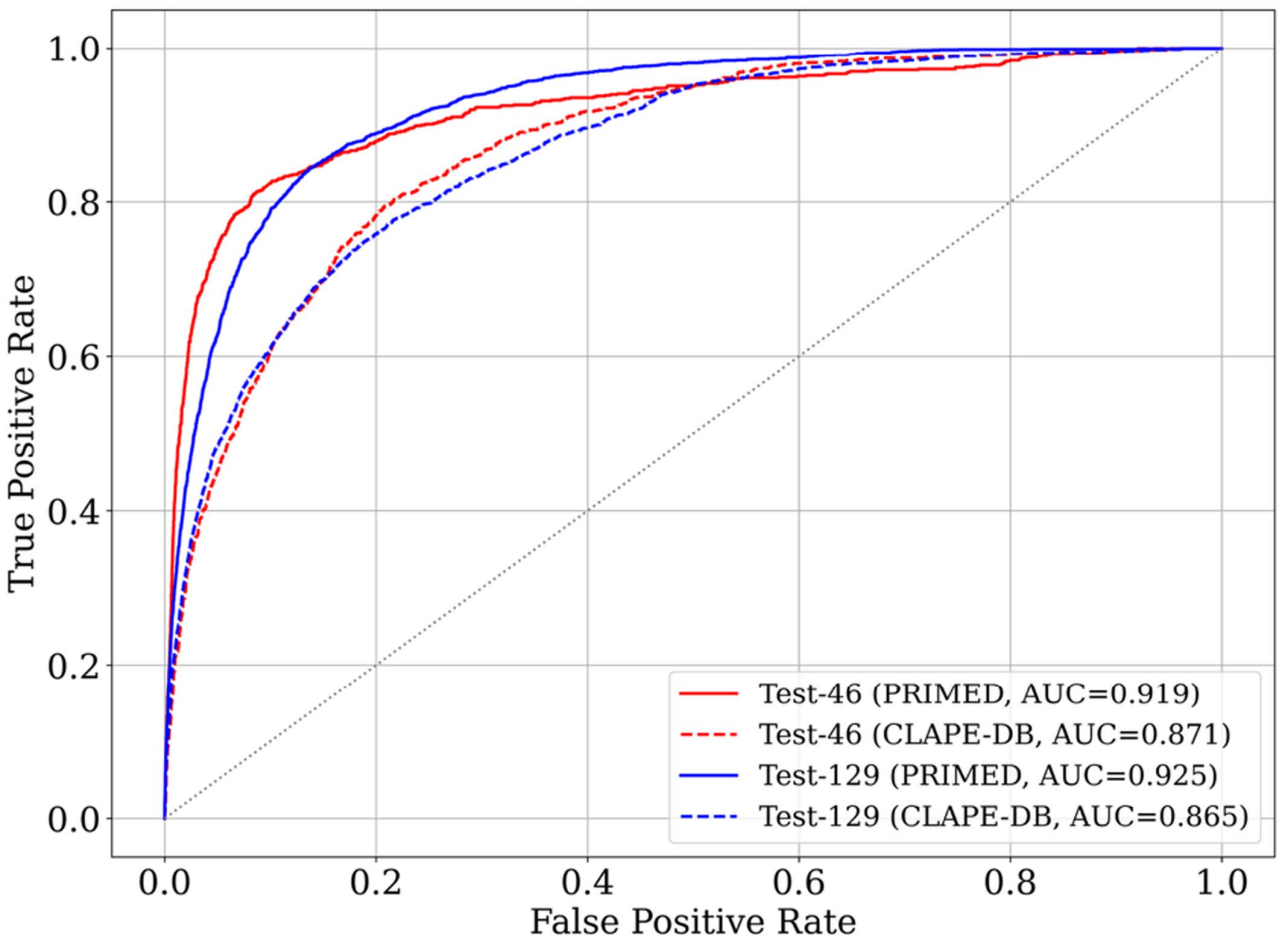
ROC curves for PRIMED and CLAPE-DB on Test-46 and Test-129.

As shown in Figure 3, the ROC curves provide a clear visualization of the performance difference between PRIMED and CLAPE-DB. Across both Test-46 and Test-129, PRIMED maintains a consistently higher true positive rate over nearly the entire false positive rate spectrum. PRIMED demonstrates a sharper rise in sensitivity within the low-FPR region (FPR < 0.1), a region that is especially important for high-confidence residue-level annotation. This behavior indicates that PRIMED is more effective at capturing true DBRs while minimizing false positives, complementing the quantitative improvements reported in Table 5 and reinforcing its reliability across diverse protein sequences.

**Figure 4:**
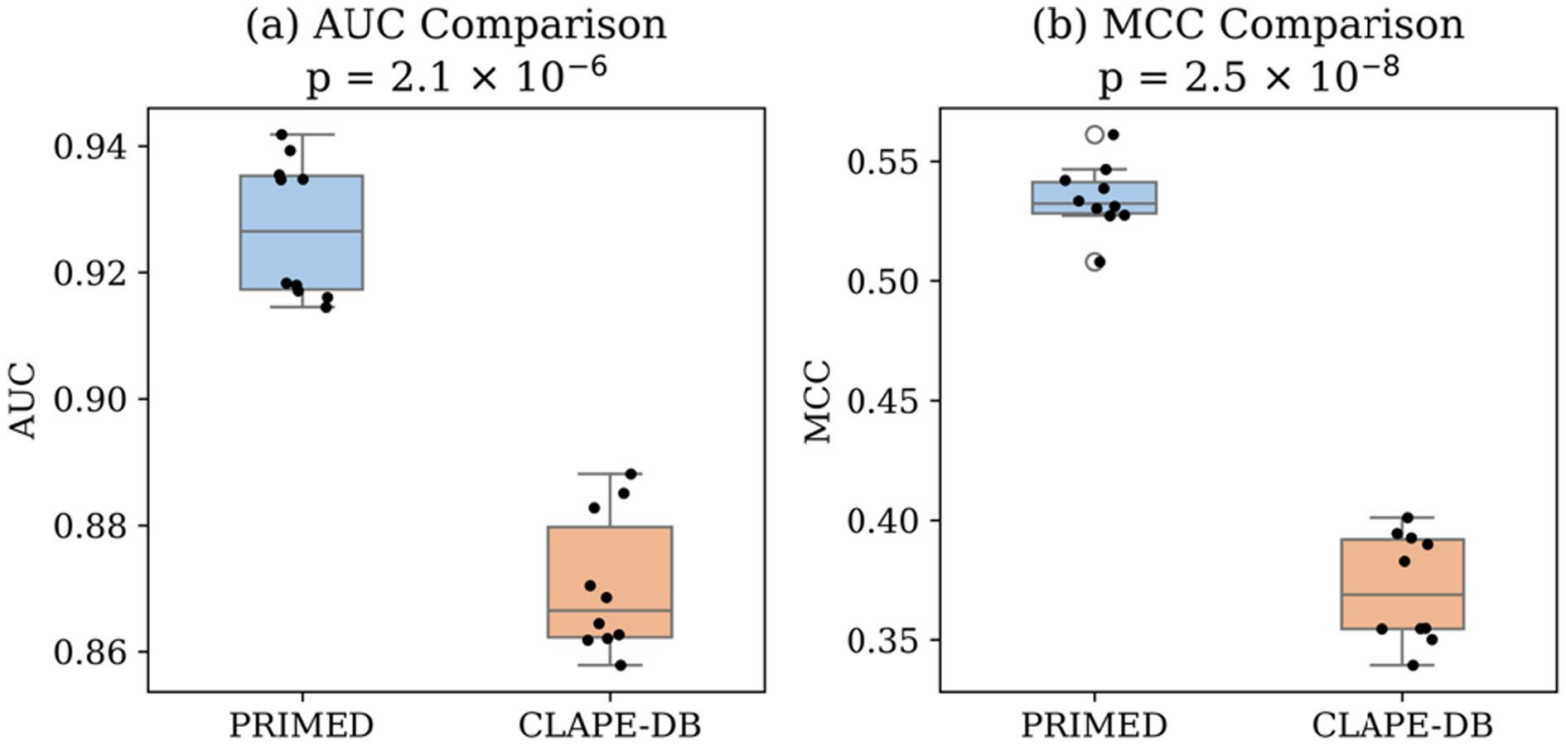
**(a)** Boxplot of AUC scores for ten sampled protein subsets, comparing PRIMED to CLAPE-DB. **(b)** Boxplot of MCC scores from the same protein subsets. Each dot represents the per-subset prediction performance (sampled at 50% of the combined Test-46 and Test-129 benchmark datasets).

Additionally, we performed a statistical analysis, combining the two test datasets. As shown in Figure 4, PRIMED significantly outperformed CLAPE-DB across both metrics. The average AUC improvement was statistically significant (*p*-value = 2.1 × 10^−6^), and the MCC comparison also showed a highly significant difference (*p*-value = 2.5 × 10^−8^). These results confirm that the improvements observed in overall model performance (Section 3.2) are robust and statistically reliable.

**Figure 5:**
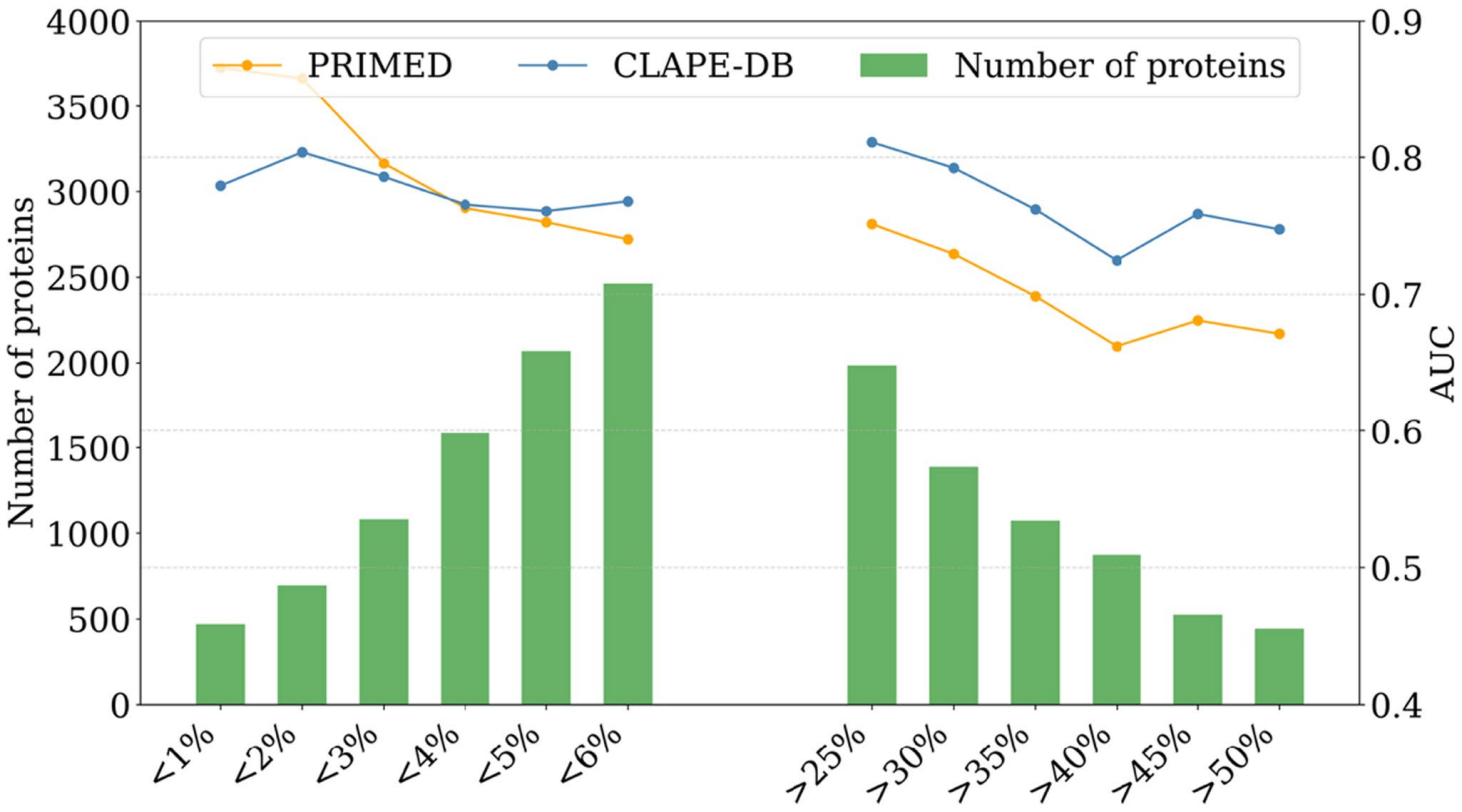
Performance of PRIMED and CLAPE-DB across protein sets with varying DNA-binding residue content on Test-10 K. The AUC value is shown as the performance metric. Green bars indicate the number of proteins in each testing set.

On the datasets curated from the UniProt database (Test-10 K), we evaluated the model generalizability of PRIMED and CLAPE-DB across diverse evaluation sets with different DBR percentages (e.g., <1, <2%, …, >25%, …, >50%). Overall, both models exhibit reduced AUC values on the Test-10 K dataset compared to the PDB-based test datasets. Such a phenomenon reflects different DBR ascertainment and/or sequence diversity in the UniProt database. Figure 5 shows that PRIMED consistently outperforms CLAPE-DB for proteins with low DBR content (<6%), highlighting its high specificity. In proteins with high DBR content (>25%), CLAPE-DB shows stronger performance, consistent with its high sensitivity. These results suggest the importance of harmonizing the definition of DBR and improving the generalizability of computational models.

In addition, PRIMED applies a threshold optimized for MCC during training (0.813), which balances sensitivity and specificity under class imbalance. However, this threshold can be adjusted depending on application needs. For example, lowering it to increase sensitivity at the cost of specificity may be desirable in contexts where missing DBRs is more detrimental than including false positives. This flexibility allows PRIMED to adapt to different prioritization strategies in downstream tasks. Reducing PRIMED’s threshold to 0.5 improves sensitivity at the expense of classification balance, resulting in an MCC of 0.311 on the full Test-10 K dataset. In comparison, CLAPE-DB achieves an MCC of 0.353 at its predefined threshold of 0.207, while PRIMED attains an MCC of 0.298 at its own optimized threshold of 0.813. This illustrates the inherent trade-off between sensitivity and specificity introduced by threshold adjustments.

### 3.3 Interpretation of pLM representations

**Figure 6:**
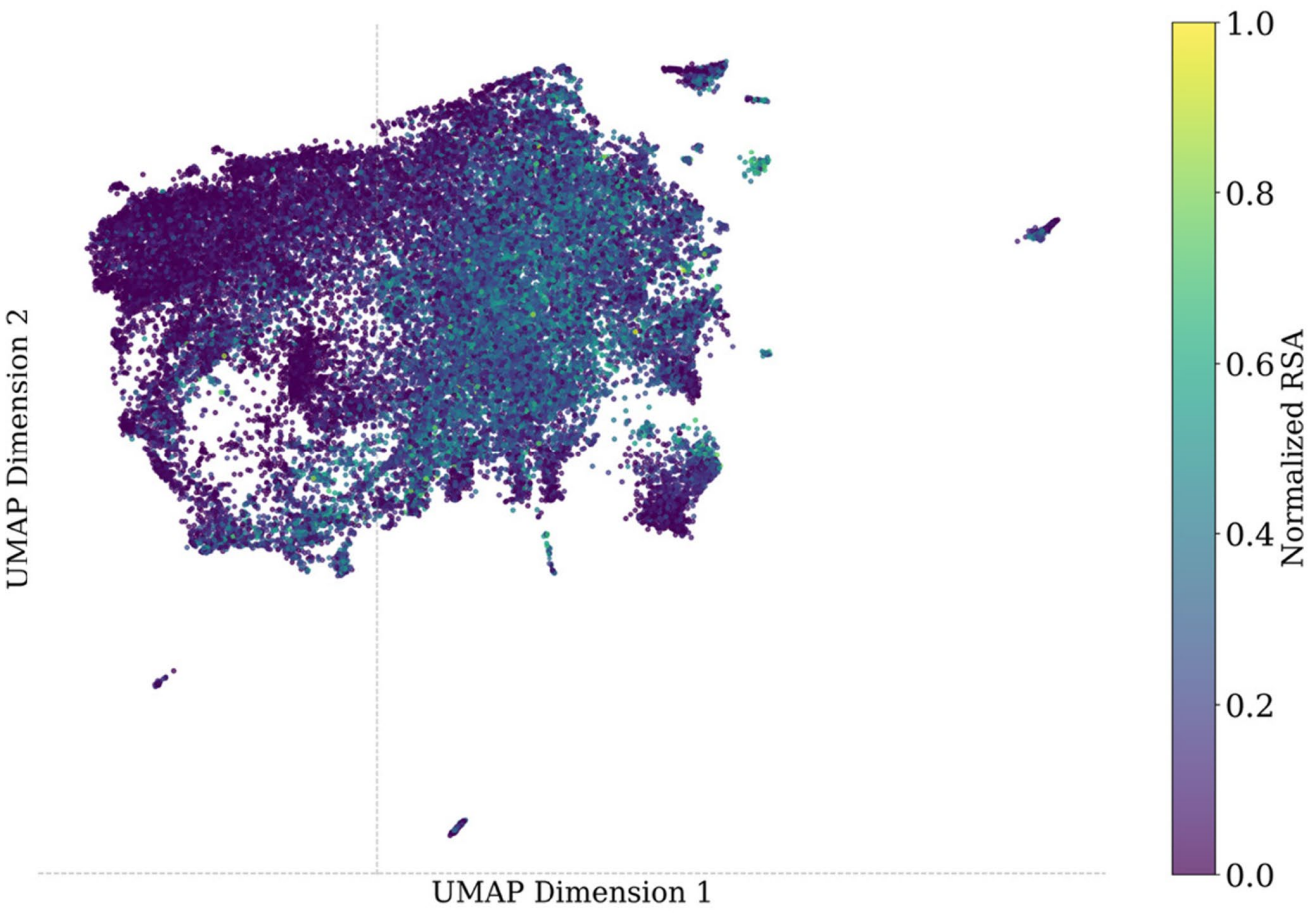
UMAP visualization of residue-level protein representations colored by RSA scores. Each dot represents a single amino acid from a protein in Test-129, projected from PRIMED input features into 2D space using UMAP. Color indicates the RSA value, mapped via the *viridis* colormap (purple: buried; yellow: exposed).

The UMAP plot in Figure 6 reveals a separation between solvent-exposed (high RSA, colored yellow) and buried (low RSA, colored blue) residues. UMAP1 exhibited a moderate correlation with RSA (Pearson’s *r* = 0.41), suggesting that the first UMAP component partially reflects variation in residue-level structural exposure. This correlation confirms that pre-trained protein language models capture biologically meaningful structural patterns associated with protein interactions.

### 3.4 Case studies

To illustrate the performance of our model, we visualized residue-level DBR predictions for two representative protein-DNA complexes: PDB IDs 4 K96 (Gao et al., 2013) and 4HQX (Davies et al., 2012). 4 K96 represents the crystal structure of the murine cyclic GMP-AMP synthase bound to double-stranded DNA, a key innate immune sensor that triggers type I interferon responses. The protein-DNA interface in 4 K96 is biologically critical for the activation of DNA sensing and signaling. 4HQX depicts the crystal structure of human PDGF-BB in complex with a modified DNA aptamer, highlighting a hormone-DNA interaction interface relevant to regulatory signaling pathways. For each protein, we compared DNA-binding scores predicted by PRIMED and CLAPE-DB against the ground truth.

**Figure 7:**
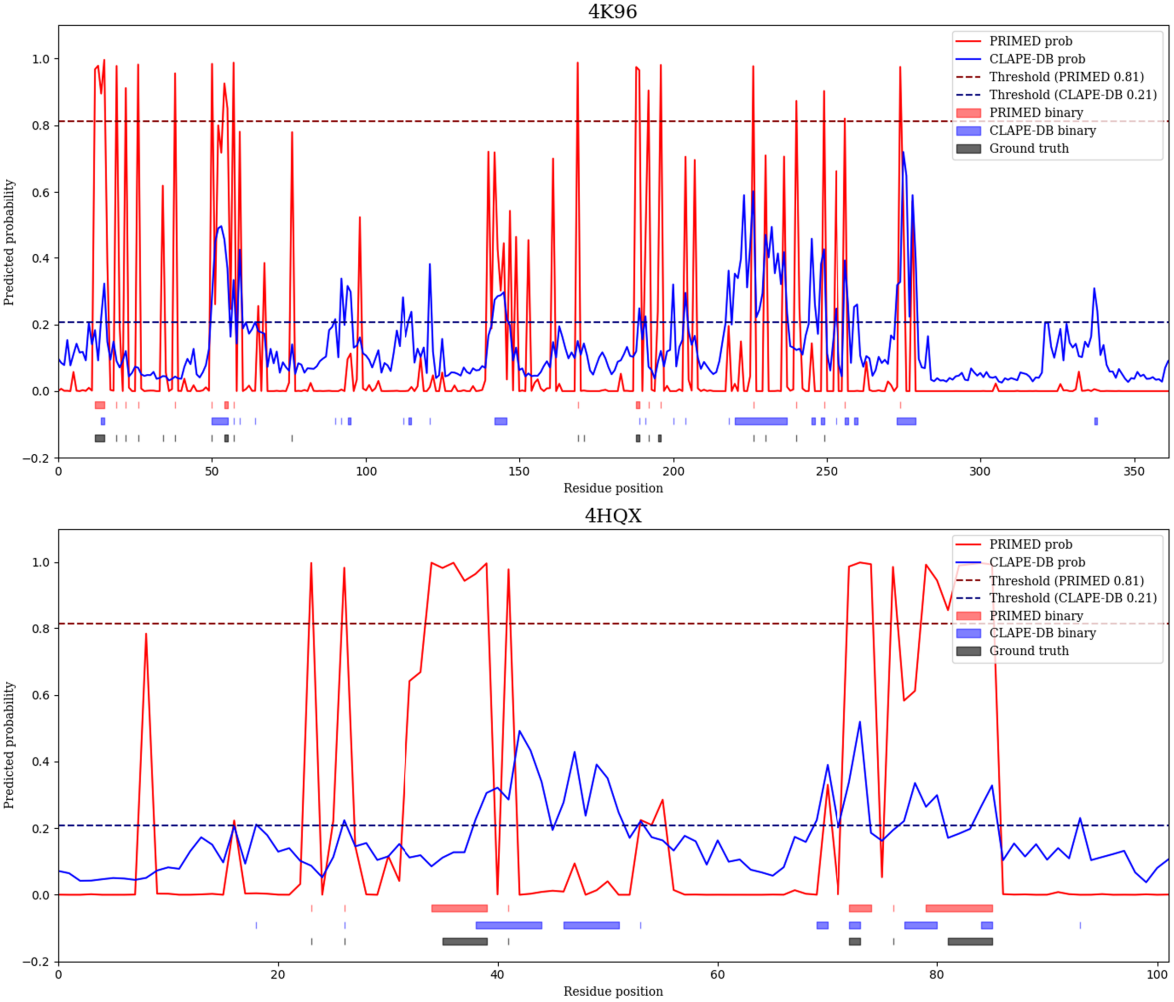
Predicted DNA-binding residues from PRIMED (red) and CLAPE-DB (blue) are shown along with experimentally annotated DNA-binding sites (black bars). The examples include 4 K96 from *Mus musculus* and 4HQX from *Homo sapiens*. Dashed horizontal lines indicate the model-specific classification thresholds used to binarize predictions. In both proteins, PRIMED aligns more closely with the ground-truth annotations than CLAPE-DB.

As shown in Figure 7, the predicted probability profiles produced by PRIMED (red curves) more accurately align with real DNA-binding regions (indicated by black ground truth bars), while CLAPE-DB (blue curves) often overpredicts binding regions. Notably, our model captures DBRs in multiple regions that CLAPE-DB fails to identify.

## 4 Discussion

In this study, we developed a machine learning framework named PRIMED that integrates concatenated protein representations from three pLMs: ESM-2, ESM-3, and ESM-C, to predict DBRs. Our model, using a simple MLP classifier, achieved significant improvements in prediction performance and robustness across three independent benchmark datasets (Test-46, Test-129, Test-10 K). The ability to generalize across datasets suggests that our methodology captures transferable biological signals, surpassing recent benchmarks and reinforcing the shift toward self-supervised learning in bioinformatics.

Our findings align with the growing body of work leveraging pLMs to predict protein structure and function. In addition, UMAP-based projection demonstrated the biochemical context carried by the pLM representations. These results underscore the value of pLMs, offering expressive and scalable representations for fine-grained protein structural and functional predictions.

This work also benefits from the use of high-quality benchmark datasets, rigorous sample-redundancy removal, and consistent separation between the training and evaluation datasets. However, the lack of a unified definition of DBRs remains a major challenge in the field. Methods trained under one definition often fail to generalize to DBRs defined differently. Moreover, residues lacking DBR annotations are typically treated as non-binding, leading to inflated false negatives. We therefore advocate establishing a gold-standard definition of DBRs and developing more carefully curated benchmarks for training and testing computational methods.

Our results open new possibilities for high-throughput annotation of DBRs across diverse organisms. In conclusion, this study presents a scalable, sequence-based framework that achieves state-of-the-art accuracy in predicting DBRs using pre-trained pLM representations as features. By leveraging the pLMs and an MLP architecture, our approach balances interpretability, efficiency, and predictive power. These findings support the broader utility of pLM-derived protein representations in biological sequence analysis and highlight promising avenues for future work in protein structural and functional predictions.

## Data Availability

Publicly available datasets were analyzed in this study. This data can be found here: https://github.com/luoshu-zhang/PRIMED/tree/main/datasets.
