# Supplementary material for: PRIMED: predicting DNA binding residues by leveraging pre-trained protein language models
Source: Front Artif Intell. 2026 Mar 25;9:1763313. doi: 10.3389/frai.2026.1763313 (PMC13056883; doi:10.3389/frai.2026.1763313)
Supplement: Supplementary file 1 [file Data_Sheet_1.docx]

## **Supplementary Materials**

**Supplementary** **Table S1.** Amino acid enrichment and depletion at DNA-binding residues in the Test-129 dataset. Residue frequencies are reported as percentages among DNA-binding residues (DBR%) and among all residues in the dataset (Background%). Enrichment ratios were computed as DBR% / Background%, and residues are sorted by enrichment ratio in descending order.

| **Residue** | **DBR (%)** | **Background (%)** | **Enrichment** |
| --- | --- | --- | --- |
| R | 15.67 | 5.86 | **2.68** |
| K | 14.33 | 7.43 | **1.93** |
| T | 8.08 | 5.10 | 1.59 |
| Y | 5.85 | 3.71 | 1.58 |
| H | 3.04 | 2.22 | 1.37 |
| S | 8.17 | 6.09 | 1.34 |
| N | 5.58 | 4.58 | 1.22 |
| W | 1.47 | 1.21 | 1.21 |
| Q | 4.69 | 3.90 | 1.20 |
| G | 6.03 | 5.24 | 1.15 |
| M | 2.05 | 2.16 | 0.95 |
| P | 2.50 | 4.05 | 0.62 |
| A | 4.15 | 6.83 | 0.61 |
| F | 2.63 | 4.45 | 0.59 |
| V | 3.35 | 6.06 | 0.55 |
| C | 0.71 | 1.43 | 0.50 |
| D | 2.63 | 5.45 | 0.48 |
| E | 2.77 | 7.16 | **0.39** |
| L | 3.97 | 10.65 | **0.37** |
| I | 2.32 | 6.41 | **0.36** |

**Supplementary Table S2.** Amino acid enrichment and depletion at DNA-binding residues in the Test-46 dataset.

DBR (%) represents the proportion of each amino acid among DNA-binding residues, and Background (%) represents its proportion among all residues in the dataset. Enrichment ratios (DBR% / Background%) are shown, with residues ordered by decreasing enrichment.

| **Residue** | **DBR (%)** | **Background (%)** | **Enrichment** |
| --- | --- | --- | --- |
| R | 13.47 | 5.58 | **2.41** |
| W | 2.28 | 1.20 | **1.91** |
| K | 13.58 | 7.26 | **1.87** |
| Y | 5.28 | 3.01 | 1.76 |
| T | 7.67 | 5.17 | 1.48 |
| N | 6.01 | 4.53 | 1.33 |
| Q | 5.70 | 4.44 | 1.28 |
| S | 8.70 | 6.87 | 1.27 |
| H | 3.42 | 3.37 | 1.01 |
| F | 4.04 | 4.02 | 1.01 |
| G | 4.04 | 5.92 | 0.68 |
| P | 3.21 | 4.80 | 0.67 |
| M | 1.45 | 2.28 | 0.64 |
| E | 4.15 | 6.79 | 0.61 |
| I | 3.21 | 5.50 | 0.58 |
| A | 3.94 | 7.38 | 0.53 |
| C | 0.83 | 1.61 | 0.52 |
| L | 4.77 | 9.62 | 0.50 |
| D | 1.97 | 4.87 | **0.40** |
| V | 2.28 | 5.77 | **0.39** |

**Supplementary** **Table S3.** Amino acid enrichment and depletion at DNA-binding residues in the Test-10K dataset. Amino acid composition was quantified for DNA-binding residues (DBR%) and for all residues in the dataset (Background%). The enrichment ratio, defined as DBR% divided by Background%, reflects relative residue preference at DNA-binding sites. Residues are sorted by enrichment ratio.

| **Residue** | **DBR (%)** | **Background (%)** | **Enrichment** |
| --- | --- | --- | --- |
| W | 1.77 | 1.04 | **1.70** |
| C | 2.36 | 1.48 | **1.59** |
| R | 9.37 | 5.94 | 1.58 |
| K | 8.57 | 5.85 | 1.46 |
| Y | 3.28 | 2.72 | 1.21 |
| F | 3.93 | 3.31 | 1.19 |
| I | 5.09 | 4.82 | 1.06 |
| V | 5.70 | 5.54 | 1.03 |
| H | 2.60 | 2.60 | 1.00 |
| T | 5.28 | 5.40 | 0.98 |
| N | 4.28 | 4.40 | 0.97 |
| E | 6.36 | 6.75 | 0.94 |
| L | 8.48 | 9.21 | 0.92 |
| Q | 4.64 | 5.07 | 0.91 |
| A | 6.77 | 7.58 | 0.89 |
| G | 5.29 | 6.27 | 0.84 |
| M | 2.02 | 2.42 | 0.83 |
| S | 6.56 | 8.44 | 0.78 |
| D | 3.93 | 5.16 | 0.76 |
| P | 3.72 | 5.98 | 0.62 |

**Supplementary Table S4.** The hyperparameter selection of MLP architectures with the input feature of ESM-2 was evaluated using five-fold cross-validation on the training set. Shown are the average AUC across folds for each configuration. The best-performing setting is highlighted in bold.

| **First Layer** | **Second Layer** | **Third Layer** | **Validation AUC** |
| --- | --- | --- | --- |
| 8 | - | - | 0.943 |
| 64 | - | - | 0.949 |
| 128 | - | - | 0.950 |
| 256 | - | - | 0.951 |
| 512 | - | - | 0.951 |
| 1024 | - | - | 0.950 |
| 128 | 64 | - | 0.950 |
| 256 | 64 | **-** | 0.951 |
| 512 | 64 | - | 0.952 |
| 512 | 128 | - | 0.952 |
| **1024** | **64** | **-** | **0.954** |
| 1024 | 128 | - | 0.948 |
| 1024 | 256 | - | 0.952 |
| 1536 | 256 | - | 0.950 |
| 1024 | 128 | 8 | 0.948 |
| 1024 | 128 | 64 | 0.942 |
| 1024 | 256 | 8 | 0.948 |
| 1024 | 256 | 64 | 0.945 |
| 1024 | 256 | 128 | 0.940 |

**Supplementary Table S5.** The hyperparameter selection of MLP architectures with the input feature of ESM-3 was evaluated using five-fold cross-validation on the training set. Shown are the average AUC across folds for each configuration. The best-performing setting is highlighted in bold.

| **First Layer** | **Second Layer** | **Third Layer** | **Validation AUC** |
| --- | --- | --- | --- |
| 8 | - | - | 0.950 |
| 64 | - | - | 0.953 |
| 128 | - | - | 0.954 |
| 256 | - | - | 0.954 |
| 512 | - | - | 0.954 |
| 1024 | - | - | 0.954 |
| 128 | 64 | - | 0.952 |
| 256 | 64 | **-** | 0.955 |
| 512 | 64 | - | 0.956 |
| 512 | 128 | - | 0.955 |
| **1024** | **64** | **-** | **0.957** |
| 1024 | 128 | - | 0.957 |
| 1024 | 256 | - | 0.955 |
| 1536 | 256 | - | 0.945 |
| 1024 | 128 | 8 | 0.955 |
| 1024 | 128 | 64 | 0.953 |
| 1024 | 256 | 8 | 0.954 |
| 1024 | 256 | 64 | 0.954 |
| 1024 | 256 | 128 | 0.953 |

**Supplementary Table S6.** The hyperparameter selection of MLP architectures with the input feature of ESM-C was evaluated using five-fold cross-validation on the training set. Shown are the average AUC across folds for each configuration. The best-performing setting is highlighted in bold.

| **First Layer** | **Second Layer** | **Third Layer** | **Validation AUC** |
| --- | --- | --- | --- |
| 8 | - | - | 0.940 |
| 64 | - | - | 0.951 |
| 128 | - | - | 0.954 |
| 256 | - | - | 0.956 |
| 512 | - | - | 0.954 |
| 1024 | - | - | 0.956 |
| 128 | 64 | - | 0.955 |
| 256 | 64 | **-** | 0.957 |
| 512 | 64 | - | 0.959 |
| 512 | 128 | - | 0.957 |
| **1024** | **64** | **-** | **0.960** |
| 1024 | 128 | - | 0.957 |
| 1024 | 256 | - | 0.958 |
| 1536 | 256 | - | 0.955 |
| 1024 | 128 | 8 | 0.955 |
| 1024 | 128 | 64 | 0.952 |
| 1024 | 256 | 8 | 0.955 |
| 1024 | 256 | 64 | 0.954 |
| 1024 | 256 | 128 | 0.953 |

**Supplementary Table S7.** Performance comparison between PRIMED and single ESM representation models. Reported values are differences in AUC (ΔAUC) and MCC (ΔMCC), and *P*-values from the statistical tests. Significance levels are denoted as ns (not significant) and *** (p < 0.0001).

| **Comparison** | **Δ AUC** | ***P*-value** | **Significance** | **Δ MCC** | ***P*-value** | **Significance** |
| --- | --- | --- | --- | --- | --- | --- |
| ESM-2 vs PRIMED | +0.0076 | <0.0001 | *** | +0.0003 | 0.8890 | ns |
| ESM-3 vs PRIMED | +0.0048 | 0.0001 | *** | +0.0540 | <0.0001 | *** |
| ESM-C vs PRIMED | -0.0012 | 0.0875 | ns | +0.0502 | <0.0001 | *** |


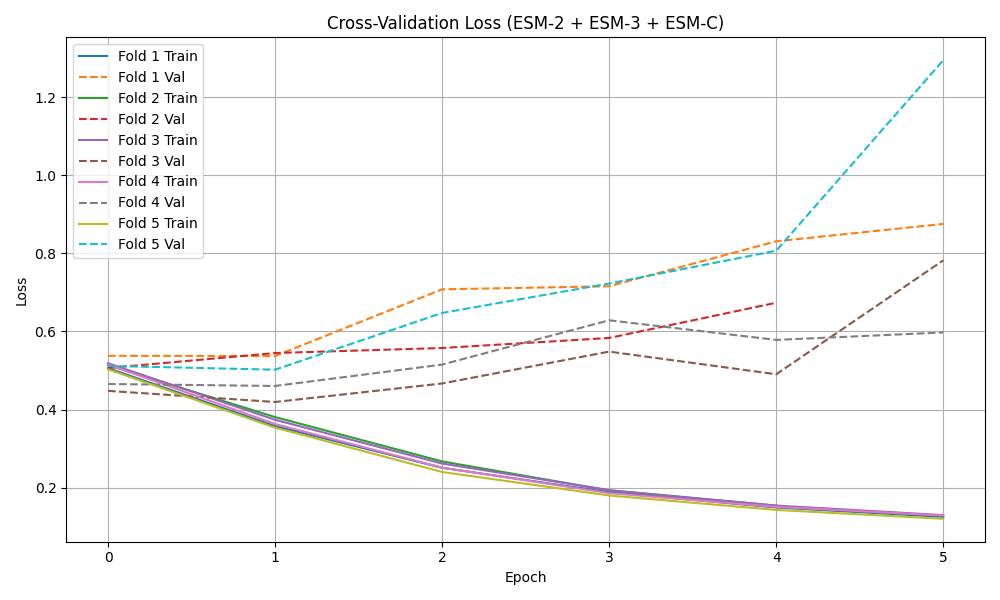


**Supplementary Figure S1.** Training curve of the PRIMED model.


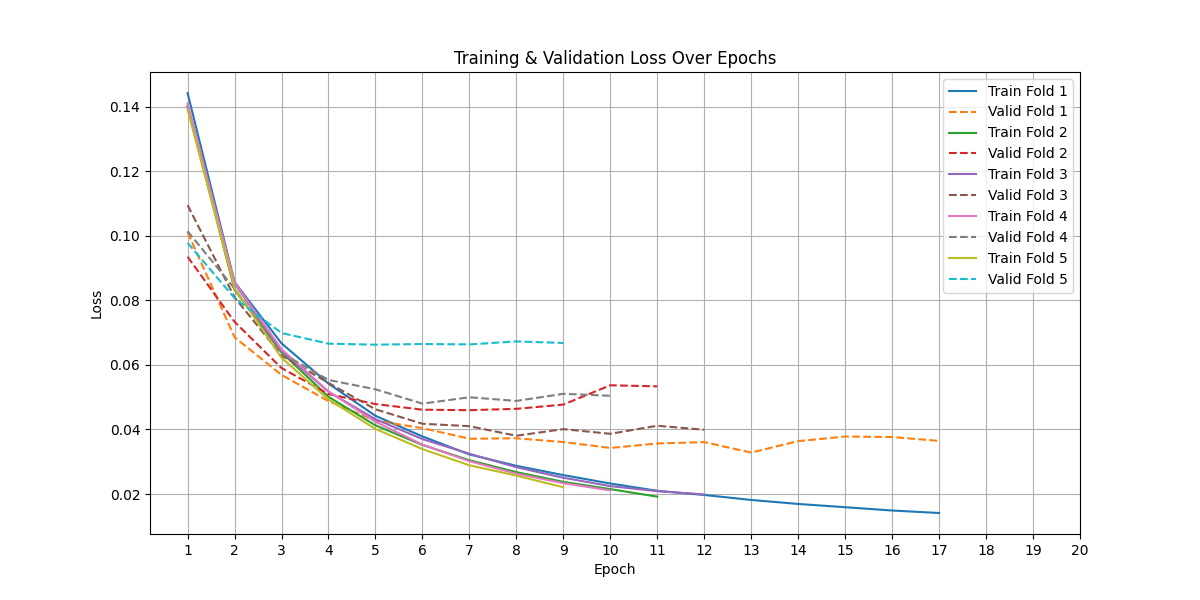


**Supplementary Figure S2.** Training curve with an input feature of ESM-2.


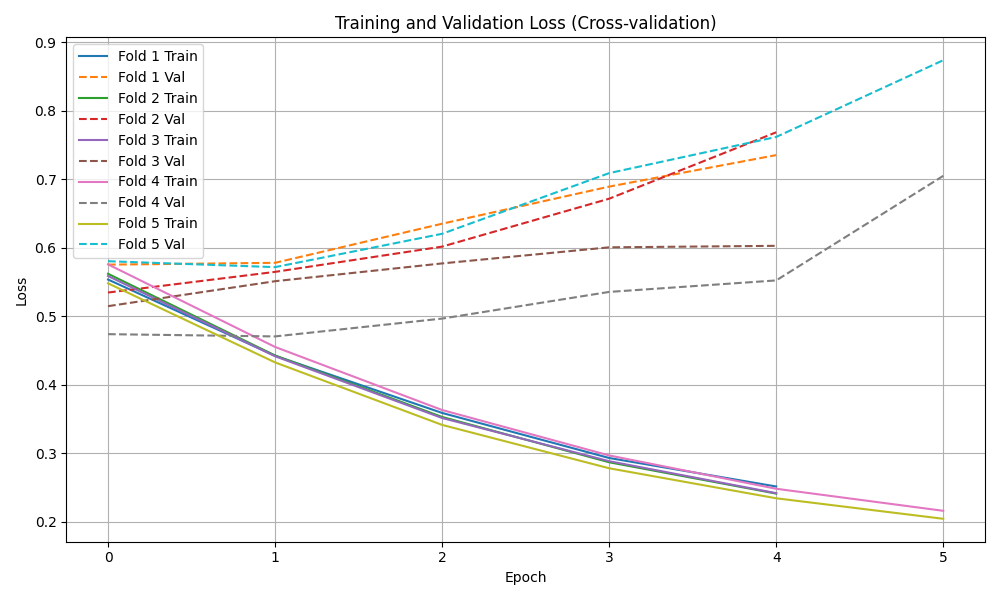


**Supplementary Figure S3.** Training curve with an input feature of ESM-3.


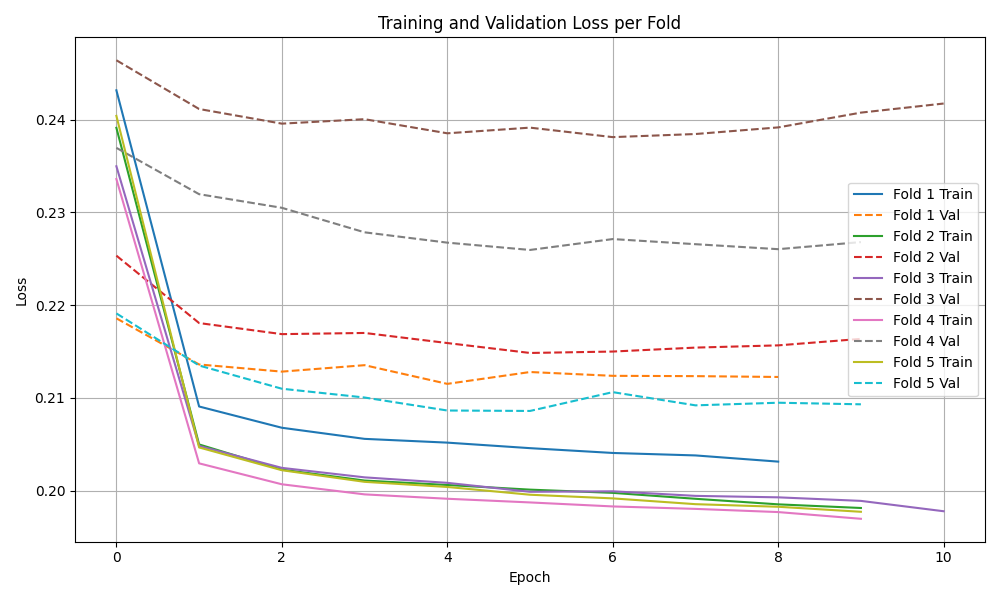


**Supplementary Figure S4.** Training curve with an input feature of ESM-C.
